# Mitochondrial DNA depletion syndrome 12B: role of photon-counting computed tomography in detection of cardiac involvement—a case report

**DOI:** 10.1093/ehjcr/ytag709

**Published:** 2026-09-19

**Authors:** Zixian Chen, Yinjia Wang, Wei Tian, Zhuoli Zhang, Gang Wang

**Affiliations:** The First Clinical Medical College of Lanzhou University, Department of Radiology, The First Hospital of Lanzhou University, Intelligent Imaging Medical Engineering Research Center of Gansu Province, Accurate Image Collaborative Innovation International Science and Technology Cooperation Base of Gansu Province, Gansu Province Clinical Research Center for Radiology Imaging, No. 1, Donggang West Road, Chengguan District, Lanzhou, Gansu 73000, China; The First Clinical Medical College of Lanzhou University, Department of Radiology, The First Hospital of Lanzhou University, Intelligent Imaging Medical Engineering Research Center of Gansu Province, Accurate Image Collaborative Innovation International Science and Technology Cooperation Base of Gansu Province, Gansu Province Clinical Research Center for Radiology Imaging, No. 1, Donggang West Road, Chengguan District, Lanzhou, Gansu 73000, China; The First Clinical Medical College of Lanzhou University, Department of Radiology, The First Hospital of Lanzhou University, Intelligent Imaging Medical Engineering Research Center of Gansu Province, Accurate Image Collaborative Innovation International Science and Technology Cooperation Base of Gansu Province, Gansu Province Clinical Research Center for Radiology Imaging, No. 1, Donggang West Road, Chengguan District, Lanzhou, Gansu 73000, China; Department of Radiological Sciences, University of California, 839 Health Sciences Road, Irvine, CA 92617, USA; The First Clinical Medical College of Lanzhou University, Department of Radiology, The First Hospital of Lanzhou University, Intelligent Imaging Medical Engineering Research Center of Gansu Province, Accurate Image Collaborative Innovation International Science and Technology Cooperation Base of Gansu Province, Gansu Province Clinical Research Center for Radiology Imaging, No. 1, Donggang West Road, Chengguan District, Lanzhou, Gansu 73000, China

**Keywords:** Mitochondrial DNA depletion syndrome, *SLC25A4*, Cardiac, Photon-counting computed tomography, Cardiac magnetic resonance, Case report

## Abstract

**Background:**

Mitochondrial DNA depletion syndrome 12B (MDS12B) is an ultra-rare autosomal recessive disorder caused by SLC25A4 mutations, which encode the mitochondrial adenine nucleotide translocator. Hypertrophic cardiomyopathy (HCM) is the predominant manifestation, yet detailed cardiac tissue characterization remains limited.

**Case summary:**

A 28-year-old male presented with a 17-year history of exertional palpitations and progressive fatigue. Echocardiography revealed severe concentric left ventricular hypertrophy with moderately reduced ejection fraction (42%). Photon-counting computed tomography (PCCT)—a novel spectral imaging technique—provided high-resolution coronary anatomy while quantifying extracellular volume expansion, late myocardial enhancement, and microvascular obstruction (MVO). Cardiac magnetic resonance (CMR) confirmed these findings. Genetic analysis identified a previously unreported homozygous SLC25A4 frameshift mutation (c.167_170del).

**Conclusion:**

This case provided the first evidence that PCCT can non-invasively detect both myocardial fibrosis and MVO in MDS12B, offering a potential alternative to CMR when contraindicated or unavailable. The novel frameshift variant expands the genotypic spectrum of *SLC25A4*-related mitochondrial depletion syndromes.

Learning points

- Photon-counting computed tomography (PCCT) non-invasively detects myocardial injury in *SLC25A4*-related mitochondrial cardiomyopathy, offering a potential alternative to CMR.
- Unlike conventional CT, PCCT offers superior spatial resolution and quantitative tissue characterization, enabling simultaneous assessment of anatomy, perfusion, and fibrosis.
- This case expands the genotypic spectrum of *SLC25A4*-related mitochondrial DNA depletion syndrome and underscores the utility of multimodal imaging.

## Introduction

Mitochondrial DNA depletion syndrome 12B (MDS12B) is an *SLC25A4*-related disorder characterized by severe mtDNA depletion and hypertrophic cardiomyopathy (HCM).^1–3^ The *SLC25A4* frameshift variant has been reported with mitochondrial myopathy, but cardiac characterization was limited to echocardiography without multimodal imaging.^4,5^ Photon-counting computed tomography (PCCT) has never been applied in *SLC25A4*-related cardiomyopathy. We report the first comprehensive multimodal imaging characterization of this variant, providing novel insights into genotype-phenotype correlations in MDS12B.

## Summary figure

**Table:** 

Time point	Clinical event	Key findings
Age 11	Symptom onset	Exertional palpitations, fatigue
Age 28	Presentation	Transient visual dimming, tachycardia; Pectus carinatum, hypertelorism; Lab: LDH (389 U/L) ↑, AST (57 U/L) ↑, TnI (0.049 ng/mL) ↑ NT-proBNP (2690.0 pg/mL) ↑
Same visit	ECG	LV hypertrophy, pathological Q waves (I, aVL, V4–V6), prolonged QTc
Same visit	Echocardiography	Concentric LV hypertrophy, LVEF 42%, GLS –10.6%
Same visit	PCCT	Myocardial bridge (LAD); ECV 37.6%; LIE:multifocal intramural delayed enhancement; focal hypodense regions
Same visit	CMR	LVEDD 66 mm; Maximal end-diastolic wall thicknesses were 21 mm (septum) and 16 mm (lateral wall); LVEF 32%; LGE: intramural ventricular septum and both intramural + subendocardial regions of the LV free wall; MVO; Elevated T1/T2/ECV
Same visit	Genetic testing	SLC25A4 homozygous frameshift (c.167_170del); Familial segregation confirmed
Follow-up	Treatment	Metoprolol, sacubitril/valsartan, dapagliflozin, diuretics; Clinically stable at 3-month follow-up

AST, aspartate aminotransferase; CMR, cardiac magnetic resonance; ECG, electrocardiogram; ECV, extracellular volume fraction; GLS, global longitudinal strain; LAD, left anterior descending artery; LDH, lactate dehydrogenase; LGE, late gadolinium enhancement; LIE, late iodine enhancement; LV, left ventricular; LVEDD, left ventricular end-diastolic diameter; LVEF, left ventricular ejection fraction; MVO, microvascular obstruction; NT-proBNP, N-terminal pro–B-type natriuretic peptide; PCCT, photon-counting CT; TnI, troponin I.

## Case presentation

A 28-year-old male with a 17-year history of exertional palpitations and fatigue was initially evaluated at an outside hospital. He was diagnosed with HCM and prescribed medical therapy, which provided symptomatic relief; however, he subsequently self-discontinued treatment. One month prior to admission, he developed recurrent episodes of transient visual dimming accompanied by tachycardia, which resolved promptly after oral glucose administration. Given symptom progression, he was referred to our centre for further evaluation.

Physical examination showed normal vital signs: temperature 36.5°C, heart rate 91 bpm, respiratory rate 19 breaths/min, and blood pressure 132/76 mmHg. Height and weight were 170 cm and 49 kg, respectively. Notable findings were mild left hemithorax prominence, increased interpupillary distance, and left exotropia. Laboratory tests showed elevated lactate dehydrogenase (389 U/L; reference 120–250 U/L), aspartate aminotransferase (57 U/L; reference 15–40 U/L), troponin I (0.049 ng/mL; reference 0.010–0.023 ng/mL), creatine kinase-MB (34.0 ng/mL; reference 2.0–7.2 ng/mL), and N-terminal pro–B-type natriuretic peptide (2690.0 pg/mL; reference 300–450 pg/mL).

The electrocardiogram (ECG) demonstrated sinus rhythm with left ventricular hypertrophy (LVH). Additional findings included pathological Q waves in leads I, aVL, and V4–V6, ST-segment elevation (0.15–0.6 mV) in V1–V4, ST depression (0.1 mV) in V6, and T-wave inversion in leads II, III, aVF, V5, and V6, with QTc prolongation (*Figure 1*). Twenty-four-hour Holter monitoring revealed frequent premature ventricular contractions and episodes of non-sustained ventricular tachycardia.

**Figure 1:**
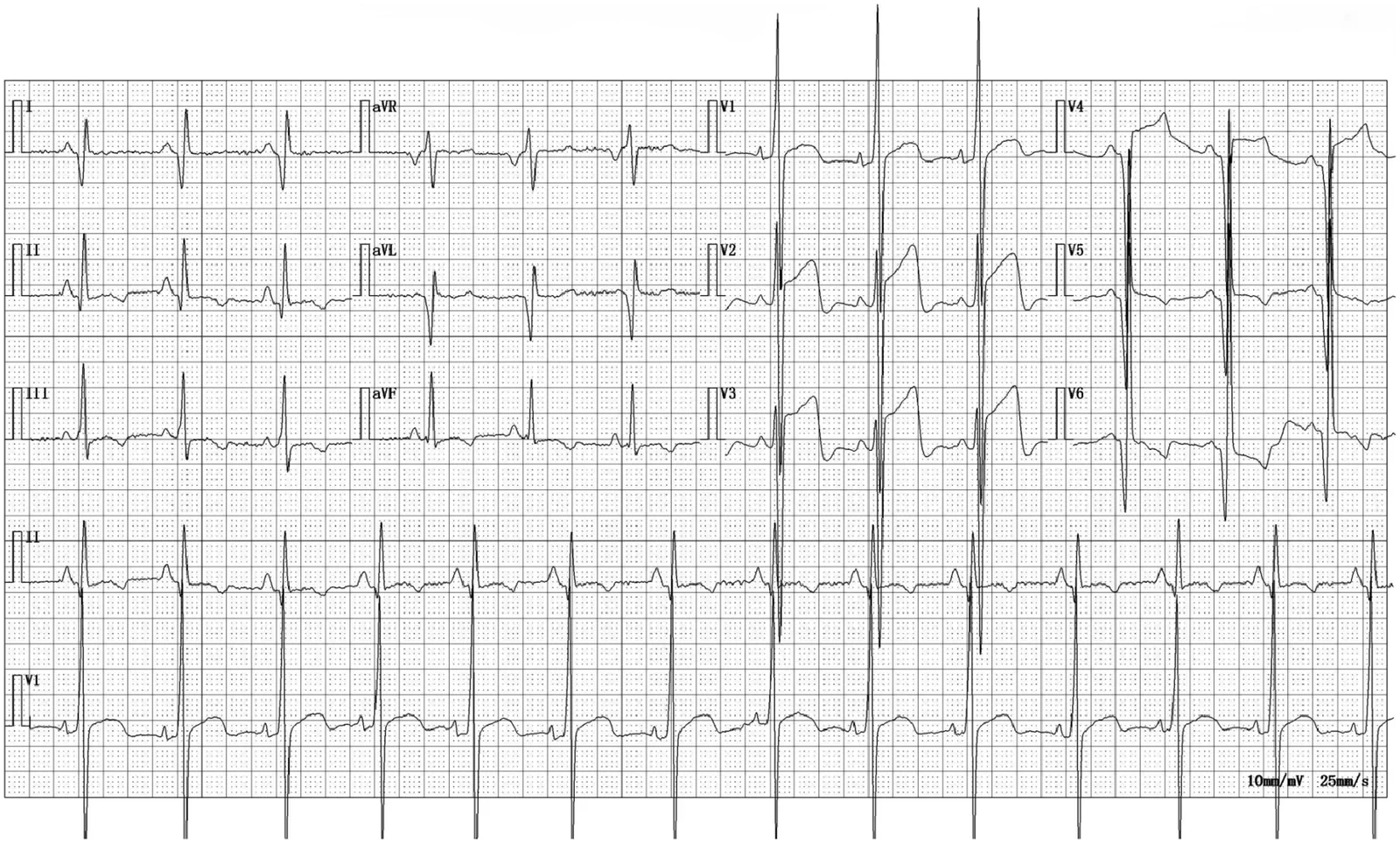
Electrocardiogram reveals sinus rhythm with left ventricular hypertrophy, pathological Q waves, ST-T segment changes, and prolonged QTc interval.

Echocardiography demonstrated left ventricular (LV) enlargement with concentric LVH and a mildly reduced left ventricular ejection fraction (LVEF) of 42%. Speckle-tracking strain analysis showed reduced strain in the anterior, inferior, anterolateral, and inferolateral segments, with a global longitudinal strain of −10.6% (*Figure 2*).

**Figure 2:**
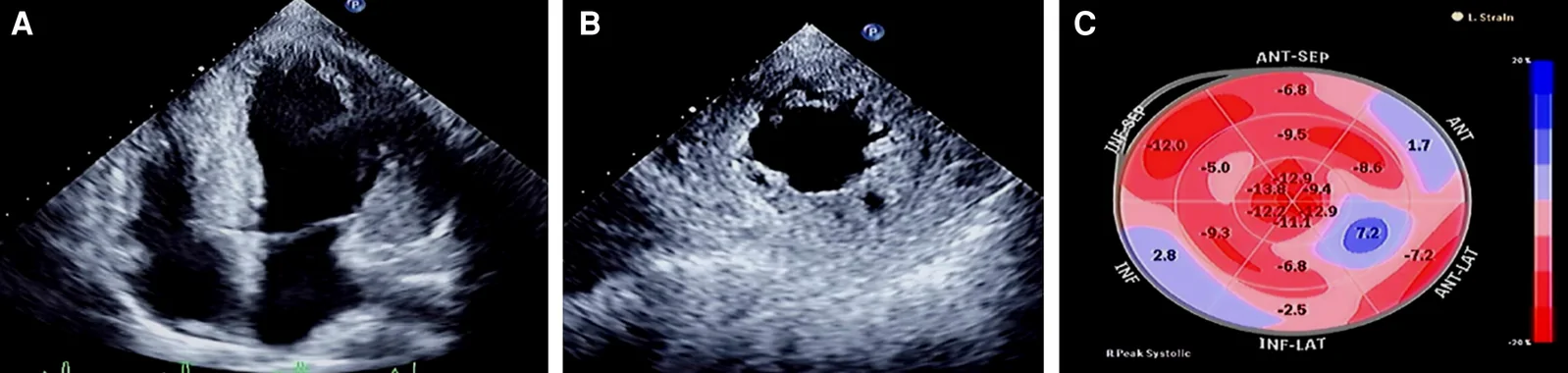
Echocardiograms findings. (*A*) Four-chamber view shows left ventricular enlargement with an end-diastolic diameter of 49 mm. (*B*) Parasternal short-axis view demonstrates concentric left ventricular hypertrophy. (*C*) Speckle-tracking strain imaging reveals impaired left ventricular global longitudinal strain (GLS, –10.6%).

Coronary CT angiography (CCTA), and cardiac magnetic resonance (CMR) were performed for cardiovascular evaluation. CCTA performed on a PCCT system revealed a myocardial bridge in the mid left anterior descending artery (*Figure 3A, 3B*), without significant systolic luminal stenosis. A curvilinear fat-density focus was noted along the LV lateral wall on calcium score images (*Figure 3C*). Late iodine enhancement (LIE) images demonstrated diffuse LV hypertrophy with multifocal intramural delayed enhancement, suggestive of myocardial fibrosis, along with focal non-enhancing hypodense suggestive of possible MVO within the interventricular septum (*Figure 3D, 3E*). The extracellular volume fraction (ECV) was elevated at 37.6% (*Figure 3F*).

**Figure 3:**
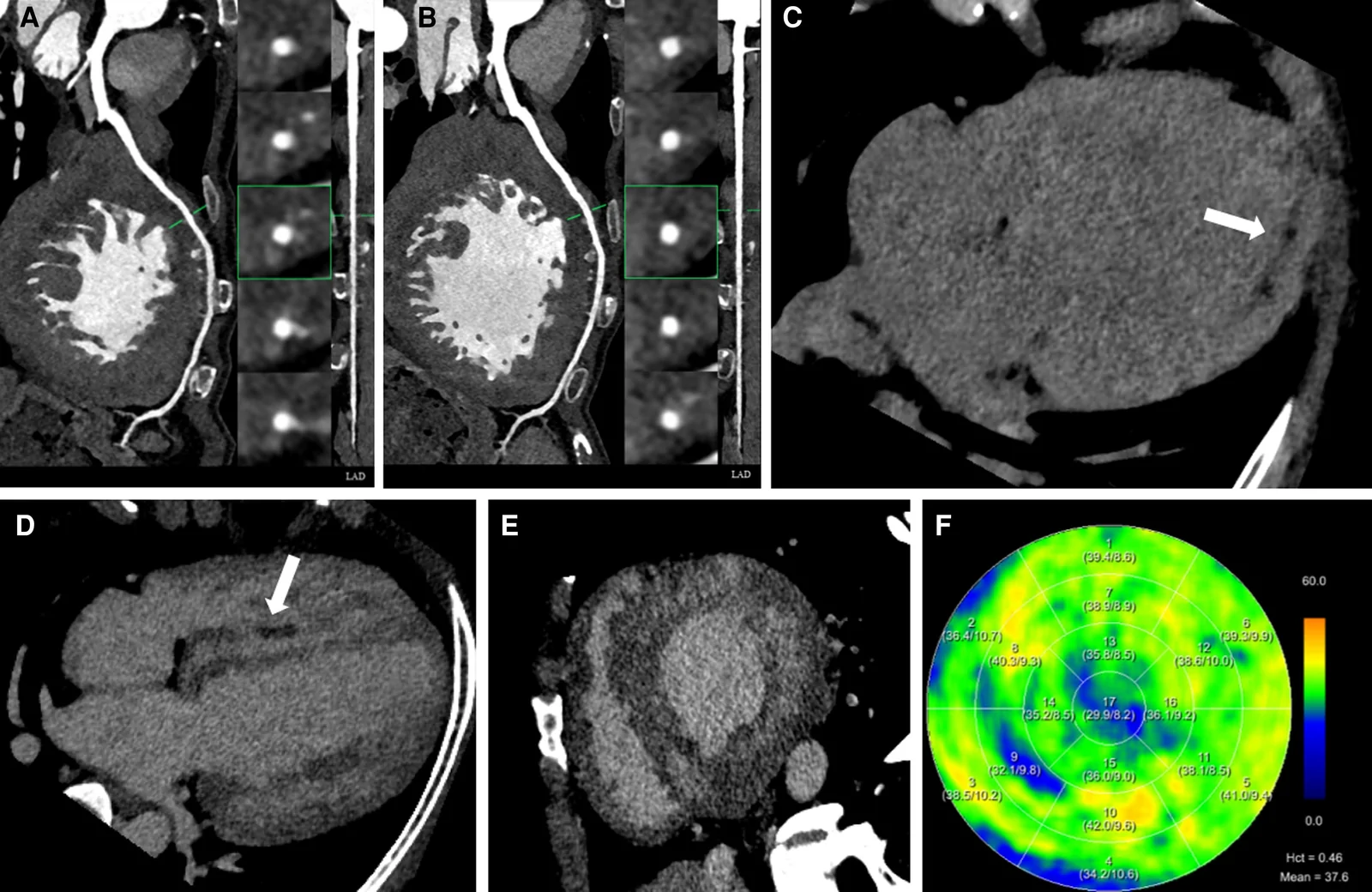
Photon-counting computed tomography images findings. (*A*, *B*) Curved planar reformation images in systole and diastole show a myocardial bridge in the mid-segment of the left anterior descending coronary artery. (*C*) Calcification score imaging demonstrates a focus of fat attenuation (arrow) in the lateral wall of the left ventricle. (*D*, *E*) Late iodine enhancement images (four-chamber and basal short-axis views) reveal concentric left ventricular hypertrophy with intramyocardial delayed enhancement; the arrow in D indicates microvascular obstruction. (*F*) The extracellular volume fraction map shows an elevated extracellular volume fraction (37.6%).

CMR confirmed LV enlargement (end-diastolic diameter 66 mm), a moderately reduced LVEF of 32%, and concentric LVH, with maximal end-diastolic wall thicknesses of 21 mm at the interventricular septum and 16 mm at the lateral wall (*Figure 4A, 4B*). Fat-phase imaging from a modified Dixon sequence identified intramyocardial fat signal within the lateral wall (*Figure 4C*). Late gadolinium enhancement (LGE) was observed in the intramural ventricular septum (basal to apex) and in both the intramural and subendocardial regions of the LV free wall (*Figure 4D–4F*). A focal non-enhancing region in the septal mid-myocardium on early gadolinium enhancement images, consistent with MVO, showed subsequent delayed enhancement on LGE images (*Figure 4G–4I*).

**Figure 4:**
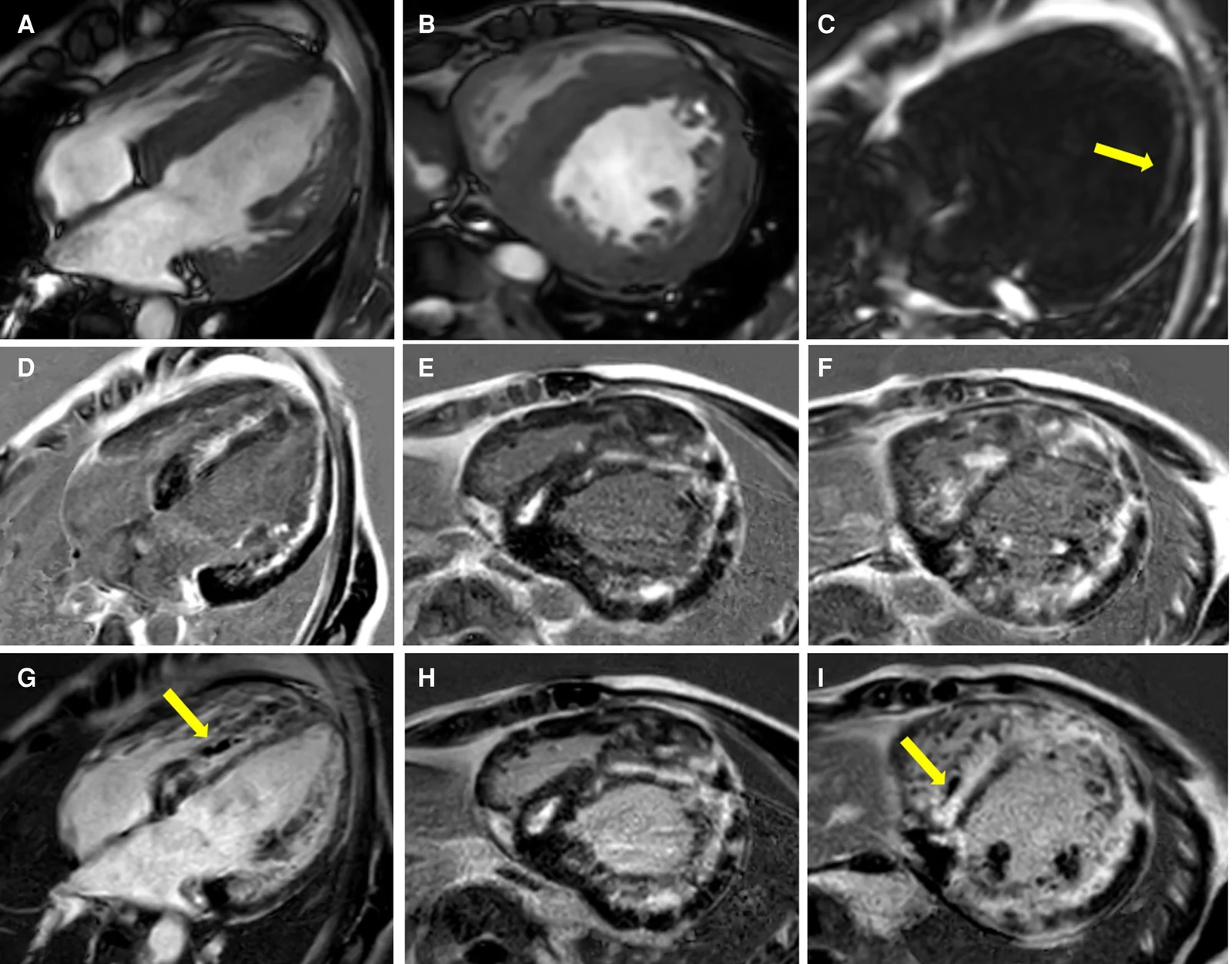
Images from cardiac magnetic resonance. (*A*, *B*) End-diastolic four-chamber and mid-ventricular short-axis balanced steady-state free precession cine images show concentric left ventricular hypertrophy (maximal wall thickness: interventricular septum, 20 mm; lateral wall, 16 mm) and a left ventricular end-diastolic diameter of 66 mm. (*C*) Fat-phase modified Dixon sequence demonstrates intramyocardial fat signal (arrow) in the left ventricular lateral wall. (*D–F*) Late gadolinium enhancement images (four-chamber, basal short-axis, and mid-ventricular short-axis views) reveal patchy intramyocardial enhancement in the interventricular septum and a circumferential late gadolinium enhancement pattern extending from the mid-ventricle to the apex along the left ventricular free wall. (*G–I*) Corresponding gadolinium enhancement images acquired within 3–5 min after first-pass perfusion reveal probable microvascular obstruction (arrows).

Tissue characterization using Cvi42 software (Circle Cardiovascular Imaging, Calgary, Canada) showed diffusely elevated native T1 (mean 1397 msec) (see Supplementary material online, Figure *S1A*) and mildly elevated T2 values in all segments except the basal and mid anterolateral walls (see Supplementary material online, Figure *S1B*), suggesting mild myocardial oedema. Myocardial ECV was globally elevated (see Supplementary material online, Figure *S1C*). Institutional reference ranges are 1250 ± 40 msec for native T1 and 50 ± 2 msec for T2 value.

Additionally, imaging findings indicated left-sided thoracic prominence and features consistent with straight back syndrome. On black-blood images, the anteroposterior-to-transverse thoracic diameter ratio was reduced to 0.29 (normal threshold >0.33), accompanied by loss of the normal thoracic kyphotic curvature (see Supplementary material online, Figure *S2*).

Based on the patient's clinical presentation and multimodal imaging findings, the diagnosis was consistent with LVH phenotype cardiomyopathy. Whole-exome sequencing identified a homozygous pathogenic *SLC25A4* frameshift mutation (c.167_170del), absent in population databases. Familial segregation analysis (*Table 1*) and database evidence classified it as pathogenic (ACMG score: 10/10).

**Table 1:** Genetic analysis of family members

Family Member	Genotype	Clinical Status	Sanger sequencing chromatogram
Patient	Homozygous c.167_170del	Symptomatic	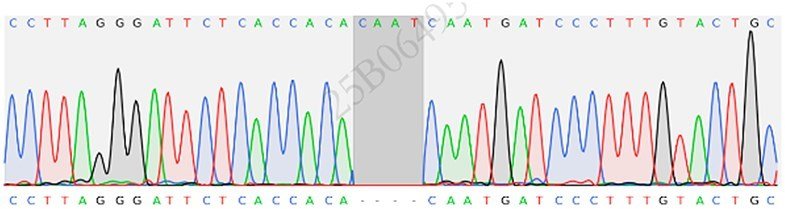
Father	Heterozygous c.167_170del	Asymptomatic carrier	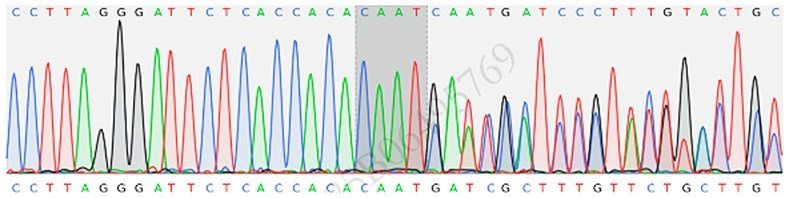
Mother	Heterozygous c.167_170del	Asymptomatic carrier	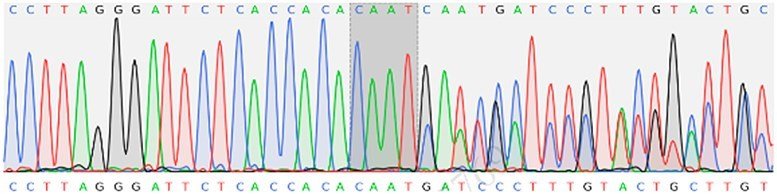
Sister	Heterozygous c.167_170del	Asymptomatic carrier	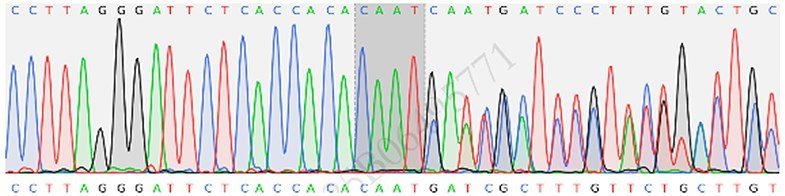

Pharmacotherapy for heart failure was initiated with metoprolol succinate, sacubitril/valsartan, dapagliflozin, furosemide, and spironolactone, consistent with guideline-directed medical therapy. Potassium citrate was added for concurrent metabolic acidosis related to mitochondrial disease. At discharge, the patient was symptomatically stable.

However, at 3-month follow-up, despite continued guideline-directed medical therapy, the patient exhibited persistent systolic dysfunction, with repeat echocardiography showing an LVEF of 34%.

## Discussion

This case report highlights two distinct but equally important aspects: (1) the clinical and genetic characterization of an ultra-rare mitochondrial cardiomyopathy due to a novel *SLC25A4* mutation and (2) the potential utility of PCCT for myocardial tissue characterization beyond conventional coronary assessment.

## Clinical and genetic features of mitochondrial DNA depletion syndrome 12B–associated cardiomyopathy

Mitochondrial DNA depletion syndrome 12B (MDS12B) is an extremely rare autosomal recessive mitochondrial disorder caused by defects in nuclear genes responsible for mitochondrial DNA maintenance, leading to impaired oxidative phosphorylation and multisystem involvement in high-energy-demand organs.^3^ In this patient, whole-exome sequencing identified a previously unreported homozygous frameshift mutation (c.167_170del; p.Asp56ValfsTer3) with familial segregation analysis confirming autosomal recessive inheritance and identifying asymptomatic heterozygous carriers (*Table 1*). This finding expands the mutational spectrum of *SLC25A4*-related mitochondrial depletion syndromes and reinforces the established genotype-phenotype correlation.

From a cardiac standpoint, this case presents a distinctive phenotype within the MDS12B spectrum. Unlike previous reports describing non-specific cardiomyopathy, our patient demonstrated a clearly defined HCM phenotype with diffuse, symmetric myocardial thickening on multimodality imaging. Importantly, structural abnormalities were accompanied by concordant functional and morphological alterations across imaging modalities, strengthening the attribution of myocardial involvement to mitochondrial dysfunction. The concordance between clinical context and multimodal imaging phenotype adds the limited imaging evidence and diagnostic experience for this rare disorder. Detailed cardiac phenotyping of *SLC25A4*-related disease remains scarce;^3,6–10^  Supplementary material online, *Table S1* provides a systematic comparison across all reported cases, including our patient.

Intramyocardial fat was detected by CMR-based m-Dixon sequence and corroborated by PCCT fat-attenuation imaging. Although fatty replacement occurs in various cardiomyopathies, in this mitochondrial context, it likely reflects chronic energy failure and cardiomyocyte loss.^11,12^ Imaging also revealed left-sided thoracic prominence consistent with straight back syndrome—a finding not specific to *SLC25A4* mutations, though scoliosis has been reported in mitochondrial disease, possibly relating to axial muscle weakness.^11,13^ Whether mitochondrial DNA inheritance contributes to the pathogenesis of spinal deformity remains speculative.^14^

Prognosis in *SLC25A4* deficiency is poor, with progressive myocardial remodelling and systolic dysfunction typical; prior cohorts report progression to end-stage heart failure in a substantial minority.^5,7^ Strauss *et al*. demonstrated that mtDNA haplogroups modulate disease severity, with haplogroup U conferring a 67% faster progression than haplogroup H.^5^ Guideline-directed medical therapy often provides limited benefit, and early genetic diagnosis with longitudinal surveillance remains essential, though therapeutic options are largely supportive.

## Potential role of photon-counting computed tomography in myocardial tissue characterization

Beyond disease characterization, this case illustrates the emerging capability of PCCT in assessing myocardial tissue properties. Hypertrophic remodelling in mitochondrial cardiomyopathy is generally considered a compensatory response to chronic energy deficiency; however, in this case, multimodality imaging demonstrated not only diffuse hypertrophy but also regionally heterogeneous myocardial attenuation patterns. These abnormalities showed spatial correspondence between PCCT and CMR, suggesting a reproducible structural correlate across modalities.^15^ This concordance supports the interpretation that the observed imaging features represent a true myocardial phenotype rather than isolated modality-specific artefacts.

In this context, PCCT provided additional insight into myocardial structural heterogeneity. The improved spatial resolution and spectral sensitivity of PCCT enabled visualization of subtle attenuation differences that corresponded to abnormal signal regions on CMR. Although CMR remains the reference standard for myocardial tissue characterization, the parallel findings across modalities in this case suggest that PCCT may capture complementary aspects of myocardial structure—particularly in complex cardiomyopathies where tissue organization is altered at a microstructural level. These observations highlight the emerging role of PCCT in quantitative myocardial tissue characterization.^16^

Nevertheless, these observations should be interpreted as exploratory. Further studies are required to determine whether the patterns observed here represent a reproducible imaging signature of mitochondrial cardiomyopathy and to establish the clinical utility of PCCT in this setting.

## Conclusion

This report presents the clinical and imaging characteristics of a rare mitochondrial disorder manifesting as an HCM phenotype, expanding the genotypic and phenotypic spectrum of *SLC25A4*-related disease. In parallel, it demonstrates the potential utility of PCCT in cardiac evaluation beyond coronary artery assessment, showing its capacity for myocardial tissue characterization comparable to CMR. Further studies are warranted to validate the clinical role of PCCT in the characterization of myocardial diseases.

## Lead author biography


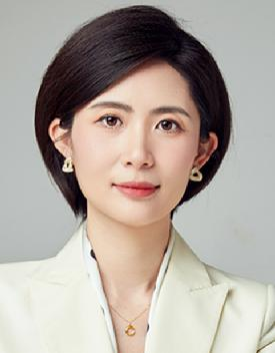
Dr. Zixian Chen is a radiologist at the First Hospital of Lanzhou University, specializing in cardiovascular disease imaging diagnosis and cardiac magnetic resonance. Dedicated to advancing diagnostic precision, her work shares CMR insights from unique cases via high-impact publications. Committed to teaching and interdisciplinary collaboration, she bridges gaps in real-world imaging-guided care.

The lead author affirms that this manuscript is an honest, accurate, and transparent account; no important aspects have been omitted.

## Supplementary Material

ytag709_Supplementary_Data

## Data Availability

All clinical details supporting this report are contained within the article.

## Notes

**Consent:** Written informed consent for the submission and publication of this case report, including the associated images and text that could potentially identify the patient, was obtained from the patient in accordance with COPE guidelines.
