# Supplementary material for: Mitochondrial DNA depletion syndrome 12B: role of photon-counting computed tomography in detection of cardiac involvement—a case report
Source: Eur Heart J Case Rep. 2026 Sep 19;10(10):ytag709. doi: 10.1093/ehjcr/ytag709 (PMC13662395; doi:10.1093/ehjcr/ytag709)
Supplement: Supplementary file 1 [file ytag709_supplementary_data.zip › Supplementary Legends Figure.docx]

**Supplementary Legends Figure 1:** Quantitative parametric mapping from CMR. (A) Native T1 map demonstrates diffusely elevated T1 values across all myocardial segments. (B) The extracellular volume (ECV) map shows increased ECV fraction globally. (C) T2 map reveals mildly elevated T2 values in most segments.

**Supplementary Legends Figure 2:** Thoracic morphology on black-blood cardiac MRI. **(A, B)** Axial and **(C)** sagittal black-blood images demonstrate left-sided prominence, a reduced anteroposterior-to-transverse thoracic diameter ratio (0.29), and straightened physiological curvature of the thoracic spine.
