# Supplementary material for: Mitochondrial DNA depletion syndrome 12B: role of photon-counting computed tomography in detection of cardiac involvement—a case report
Source: Eur Heart J Case Rep. 2026 Sep 19;10(10):ytag709. doi: 10.1093/ehjcr/ytag709 (PMC13662395; doi:10.1093/ehjcr/ytag709)
Supplement: Supplementary file 1 [file ytag709_supplementary_data.zip › Supplementary Table 1.docx]

Supplementary Table 1. Comparison of clinical and imaging phenotypes in reported *SLC25A4*-related cardiomyopathy cases

| **Patient** | **L1** | **L2** | | **L3** | **L4** | **L5** | **L6** | **L7** | **P** |
| --- | --- | --- | --- | --- | --- | --- | --- | --- | --- |
| Sex/age (years) | M/25 | F/21 | | M/28 | M/Neonatal | F/26 | F/52 | M/16 | M/28 |
| *SLC25A4* variant | c.368C > A | c.111+1G > A | | c.707G > C | c.239G>A | c.423G>C | c.368C>A | c.653A>C | c.167_170del — novel |
| HCM phenotype | + | + | | + | + | + | + | + | + |
| Skeletal symptoms | + | + | | + | + | + | + | + | SBS |
| Echocardiography | Concentric LVH, IVS:15–18 mm, LVPW: 14 mm, Non‐obstructive | LVH, IVS: 14–17 mm, LVPW: 13 mm, Non‐obstructive | | Mild hypertrophy | － | Concentric LVH, LVWT: 14 mm, LVEF > 60%, Intraventricular obstruction | Concentric LVH, LVWT: 14 mm, LVEF > 60%, Intraventricular obstruction | LVH, LVEF: 40–53%, Non‐obstructive | Concentric LVH, LVEF: 42%, GLS: –10.6%, Non‐obstructive |
| CMR findings | － | － | | － | － | － | － | － | Intramyocardial and subendocardial LGE, MVO, Elevated T1/T2/ECV, Fat |
| PCCT findings | － | － | | － | － | － | － | － | ECV: 37.6%, Multifocal intramural delayed enhancement, MVO, Fat, Myocardial bridge |
| Outcome | Compensated cardiac function | | Gradual progression | Stable | Poor prognosis | LC-treated; mild ptosis; maintained functionality | Permanent myopathy; HCM stable for years on CoQ10 | Stable | Stable on GDMT at 3 months |
| Reference | Palmer et al., 2005 | Echaniz-Laguna et al., 2012 | | Kornblum et al., 2015 | Thompson et al., 2016 | Tossem et al., 2018 (Case 1) | Tossem et al., 2018 (Case 2) | von Renesse et al., 2018 | － |

P, present study; L, literature case; +, present/positive; －, absent/negative;

LVH, left ventricular hypertrophy; IVS, interventricular septum; LVPW, left ventricular posterior wall; LVWT,

left ventricular wall thickness; LVEF, left ventricular ejection fraction; GLS, global longitudinal strain; LGE, late gadolinium enhancement; MVO, microvascular obstruction; ECV, extracellular volume fraction; LC, L-carnitine; CoQ10, Coenzyme Q10; GDMT, guideline-directed medical therapy
